# Prolactin-induced Subcellular Targeting of GLUT1 Glucose Transporter in Living Mammary Epithelial Cells

**DOI:** 10.5041/RMMJ.10223

**Published:** 2015-10-26

**Authors:** Arieh Riskin, Yehudit Mond

**Affiliations:** 1 Department of Neonatology, Bnai Zion Medical Center, Bruce & Ruth Rappaport Faculty of Medicine, Technion, Israel Institute of Technology, Haifa, Israel; 2 Section of Neonatology and ARS/USDA Children’s Nutrition Research Center, Department of Pediatrics, Baylor College of Medicine, Houston, Texas, USA; 3 Visualization Laboratory, Technion, Israel Institute of Technology, Haifa, Israel

**Keywords:** CIT3 mouse mammary epithelial cells, green fluorescent protein, GLUT1 glucose transporter, human mammary epithelial cells, prolactin

## Abstract

**Background:**

Studying the biological pathways involved in mammalian milk production during lactation could have many clinical implications. The mammary gland is unique in its requirement for transport of free glucose into the cell for the synthesis of lactose, the primary carbohydrate in milk.

**Objective:**

To study GLUT1 trafficking and subcellular targeting in living mammary epithelial cells (MEC) in culture.

**Methods:**

Immunocytochemistry was used to study GLUT1 hormonally regulated subcellular targeting in human MEC (HMEC). To study GLUT1 targeting and recycling in living mouse MEC (MMEC) in culture, we constructed fusion proteins of GLUT1 and green fluorescent protein (GFP) and expressed them in CIT3 MMEC. Cells were maintained in growth medium (GM), or exposed to secretion medium (SM), containing prolactin.

**Results:**

GLUT1 in HMEC localized primarily to the plasma membrane in GM. After exposure to prolactin for 4 days, GLUT1 was targeted intracellularly and demonstrated a perinuclear distribution, co-localizing with lactose synthetase. The dynamic trafficking of GFP-GLUT1 fusion proteins in CIT3 MMEC suggested a basal constitutive GLUT1 recycling pathway between an intracellular pool and the cell surface that targets most GLUT1 to the plasma membrane in GM. Upon exposure to prolactin in SM, GLUT1 was specifically targeted intracellularly within 90–110 minutes.

**Conclusions:**

Our studies suggest intracellular targeting of GLUT1 to the central vesicular transport system upon exposure to prolactin. The existence of a dynamic prolactin-induced sorting machinery for GLUT1 could be important for transport of free glucose into the Golgi for lactose synthesis during lactation.

## INTRODUCTION

### Biology of Milk Production and Transport Pathways of Milk Constituents

Females of all mammalians bear mammary glands, and milk secretion and lactation is a characteristic feature of all mammalian species, which are the only organisms that produce copious glandular skin secretions to feed their young.^1^,^2^ Lactation is a highly complex and evolutionarily ancient strategy of all mammals, providing their offspring with a highly digestible, concentrated, nutritionally balanced diet, while allowing adult mammals to evolve a wide range of developmental and reproductive strategies and specialize on diets that could either be too difficult to capture or digest or would be insufficient to cover the high nutritional needs of their small rapidly growing offspring.^1^–^3^ Lactation helps mammalian mothers cope with unreliable food supplies, because lactating females can draw on their nutrient reserves for milk production, suggesting an evolutionary advantage for their dependent offspring, since milk intake promotes growth, fitness, and survival of the young.^2^,^4^,^5^ Beyond nourishment of the neonate, milk also helps establish immunological and endocrine competence in the offspring. Milk’s nutrient composition varies extensively across mammalian species, as a function of evolutionary history, maternal nutrient intake, duration of milk production, and stage of lactation.^2^,^5^ Milk is a complex mixture whose composition reflects different transport and secretion mechanisms within the mammary gland that aim to answer the different nutritional needs of mammalian neonates.^6^

The lactating mammary gland is composed of branching ducts ending in alveolar clusters where milk is produced. A single layer of polarized secretory epithelial cells forms the alveolar wall. The alveoli are surrounded by myoepithelial cells and are embedded in vascularized connective tissue stroma. While growth of the mammary gland and secretion of milk are stimulated by growth hormone, prolactin, adrenocortical steroids, estrogens, and progesterone, ejection of milk requires contraction of myoepithelial cells stimulated by oxytocin.^6^,^7^ The cytoplasm of the secretory alveolar epithelial cells is filled with numerous mitochondria, extensive rough endoplasmic reticulum network, well-developed Golgi apparatus, and secretory vesicles in the apical region of the cell adjacent to the alveolar lumen. The basal side of the alveolar epithelial cells lies on a basement membrane that separates them from the stroma and vascular system. In between, epithelial cells are connected to each other by an apical complex of tight junctions that inhibit direct paracellular exchange of substances between the vascular compartment and milk in the alveolar lumen. There are five pathways by which solutes, including proteins, lipids, ions, nutrients, and water, can be transported into the milk. Four are transcellular, involving transport across at least two membrane barriers, while the fifth is paracellular and allows direct exchange of interstitial and milk components: (1) The exocytic pathway is for endogenously generated aqueous soluble substances, including lactose, oligosaccharides, the major milk proteins, citrate, phosphate, calcium, and other nutrients, which is similar to exocytic pathways in other cell types; (2) The transport pathway for milk lipids is unique to the mammary gland generating the milk fat globules; milk lipids, primarily triacylglycerides, are synthesized in the smooth endoplasmic reticulum in the basal region of the lactating alveolar cell—the newly synthesized lipid molecules are coated by protein membranes to form small storage structures called cytoplasmic lipid droplets that are transported to the apical plasma membrane, where they are secreted by a unique budding process as membrane-enveloped structures, i.e. the milk fat globules; (3) The trans-cytosis pathway transports macromolecules derived from the serum or stromal cells, including serum proteins (such as immunoglobulin, albumin, and transferrin), hormones (such as insulin, prolactin, and estrogen), and stromal-derived substances (such as secretory immunoglobulin A, cytokines, and lipoprotein lipase); (4) Various membrane transport pathways transport ions and small molecules (such as glucose, amino acids, and water) across the basal and apical plasma membranes of the polarized alveolar epithelial cell; and (5) The para-cellular pathway provides a direct route for transfer of serum and interstitial substances into the milk. These transport pathways are regulated by the functional stage of the mammary gland and by hormones and growth factors.^6^

Studying mammary gland biology, transport pathways of milk constituents, and their influence on milk production in general, and specifically investigating glucose transport mechanisms in mammary epithelial cells (MEC) that could influence lactose synthesis and thus milk volume, is important and could have many clinical implications, including in breastfeeding support to mothers, in the dairy industry, and in oncology. Breast cancer tumor cells may utilize some of the MEC transport pathways to support metabolically their rapid uncontrolled growth.^6^,^8^

### Glucose Transport Pathways in the Mammary Epithelial Cells

Human milk is rich in lactose, which is the major osmotic constituent of human milk and thus the major determinant of milk volume.^9^ Lactose is a disaccharide composed of glucose and galactose. Lactose is found only in milk and is the primary carbohydrate in milk. The final step in the biosynthesis of lactose from UDP-galactose and glucose is catalyzed by lactose synthetase, a complex of α-lactalbumin and the Golgi enzyme β1,4-galactosyltransferase.^10^ β1,4-Galactosyltransferase is embedded in the inner surface of Golgi membranes. It is membrane-bound and directed towards the lumen of the cisternal space.^10^,^11^ Galactosyltransferase is found in most tissues and is involved in protein glycosylation. In mammals β1,4-galactosyltransferase has been recruited for a second biosynthetic function, the production of lactose. This function takes place exclusively in the lactating mammary gland. Galactosyltransferase has a relatively poor affinity for glucose (*K*_m_~1 M). The affinity of this enzyme is profoundly modified by transient association with α-lactalbumin, which creates a binding site for glucose, so that the affinity of the transferase for glucose increases about 500-fold (*K*_m_~2 mM).^10^ This allows the synthesis of lactose in the Golgi to occur at the physiological concentrations found in the mammary cell. α-Lactalbumin is a milk whey protein that is not catalytically active by itself, but is necessary for the synthesis of lactose. The initiation of α-lactalbumin synthesis that occurs at parturition is required for the initiation of copious milk production, but is neither the only factor nor the limiting factor controlling lactose synthesis.^11^,^12^ Availability of glucose and UDP-galactose to the lactose synthase enzyme complex in the Golgi apparatus may be rate-limiting for lactose synthesis. Many cells possess active mechanisms for the uptake of nucleotide sugars such as UDP-galactose into the Golgi, which are essential to protein glycosylation. In contrast, the mammary gland is unique in its need to transport free glucose into the Golgi. The main known isoform of glucose transporters expressed in the mammary gland is GLUT1 (SLC2A1).^9^,^13^,^14^–^20^ Levels of GLUT1 increase progressively during pregnancy, reaching their highest levels during lactation,^15^,^16^ and this is dependent on prolactin.^21^ In most cells GLUT1 normally resides in the plasma membrane and is responsible for basal glucose uptake. In polarized epithelial cells, including mammary cells, GLUT1 is targeted primarily to the basolateral membrane.^22^ The role of GLUT1 in glucose transport into Golgi has been controversial.^15^,^23^,^24^ Evidence from *in vivo* and *in vitro* studies demonstrated unique hormonally regulated intracellular targeting of GLUT1 from the plasma membrane to a low-density intracellular compartment in mouse mammary gland during lactation.^16^,^23^–^25^ Further work distinguished this compartment from Golgi, suggesting that the hormonally induced intracellular targeting of GLUT1 in lactating MEC is into a Brefeldin A-sensitive low-density vesicle that may represent a subcompartment of *cis*-Golgi.^25^ This hormonally regulated subcellular targeting of GLUT1 may have an important role for lactose synthesis in MEC during lactation.

The aim of this work was to study GLUT1 subcellular targeting in living human and mouse mammary epithelial cells (HMEC and MMEC) in culture; and to study GLUT1 intracellular trafficking in living MMEC in culture under the effects of the lactogenic hormones that regulate it. Our hypothesis was that there would be dynamic basal trafficking of GLUT1 in living MEC that would target most GLUT1 to the basolateral membrane under maintenance conditions, and intracellularly upon exposure to prolactin, which should be the main hormonal stimulus that drives this translocation during lactation. Our studies could then complete the previous *in vitro* findings in fixed cell^25^ by adding the dynamic observations in living MEC that could in turn relate to the *in vivo* findings.^16^

## METHODS

### Cell Cultures and Media

*Human mammary epithelial cells* (Clonetics, BioWhittaker, Walkersville, MD, USA) from normal breast tissue biopsies were studied. The cells were maintained in mammary epithelial growth medium (MEGM) containing MEBM baseline medium (Clonetics, BioWhittaker, Walkersville, MD, USA), 10% inactivated fetal bovine serum (FBS), 0.3% D-glucose (Sigma, St. Louis, MO, USA), 10 ng/mL human recombinant epidermal growth factor (hEGF) (Sigma, St. Louis, MO, USA), 5 μg/mL insulin (Sigma, St. Louis, MO, USA), and 0.5 μg/mL hydrocortisone (Sigma, St. Louis, MO, USA). To stimulate differentiation by lactogenic hormones, the medium was changed to mammary epithelial secretion medium (MESM), by adding prolactin 3 μg/mL (Sigma, St. Louis, MO, USA), increasing hydrocortisone concentration to 3 μg/mL, and withdrawing hEGF. The cells were treated for 4 days in MESM before studying them.

*CIT3 cells* were kindly provided by M.C. Neville, PhD, University of Colorado School of Medicine. CIT3 cells are a non-neoplastic cell line derived from MMEC (after being selected from Comma-1-D cells for their ability to grow well on filters, form tight junctions, and exhibit polarized transport).^26^ Cells were maintained in growth medium (GM), which is a nutrient-defined basal medium (DMEM/F12) (GibcoBRL, Life Technologies Inc., Rockville, MD, USA), containing 10 μg/mL insulin and 5 ng/mL epithelial growth factor (EGF). To stimulate differentiation by lactogenic hormones, the medium was changed to secretion medium (SM), by adding prolactin 3 μg/mL and hydrocortisone 3 μg/mL, and withdrawing EGF. The routine exposure to SM was 96 hours prior to evaluating changes in GLUT1 subcellular targeting.

### Subcloning GLUT1 cDNA into Green Fluorescent Protein Plasmid Vectors

Enhanced GFP (EGFP) carries a red-shifted variant of wild-type green fluorescent protein (GFP), which has been optimized for brighter green fluorescence and higher expression in mammalian cells. It has an excitation maximum of 488 nm and emission maximum of 507 nm. Its usefulness as a fluorescent “tag” in dynamic intracellular trafficking and targeting studies was reported.^27^,^28^ Green fluorescent protein (GFP) plasmid vectors (p) pEGFP-C1 and pEGFP-N1 (#6084-1 and #6085-1, respectively, Clontech Laboratories Inc., Palo Alto, CA, USA) were used. We recovered GLUT1 cDNA^29^ from pHepG2 using Bam H1 restriction digest or, in a separate experiment, by a polymerase chain reaction (PCR). The primers for the PCR reaction were designed to include Hind III and Bam H1 restriction sites at the N- and C-terminus of GLUT1 cDNA, respectively. Restriction digest of the insert and the plasmid vector multiple cloning sites with these enzymes followed by ligation allowed GLUT1 cDNA to be subcloned in the correct orientation. We subcloned GLUT1 cDNA into pEGFP-C1 and pEGFP-N1, respectively, to create N- and C-terminus fusion of GLUT1 to GFP. The N-terminus fusion of GLUT1 to EGFP was also constructed by subcloning GLUT1 cDNA, recovered by Bam H1 restriction digest, into Bam H1 site of pEGFP-C1. This could not be done for the C-terminus fusion of GLUT1 cDNA due to an intervening stop codon. All the recombinant vectors were sequenced to verify the correct orientation and exclude mutations.

*pECFP-Golgi* encodes a fusion protein consisting of enhanced cyan fluorescent protein (ECFP) and a sequence encoding the N-terminal 81 amino acids of human β1,4-galactosyltransferase.^30^,^31^ This region of human β1,4-galactosyltransferase contains the membrane-anchoring signal peptide that targets the fusion protein to the trans-medial region of the Golgi apparatus.^32^–^34^ The ECFP fluorescence excitation maxima (433 nm and 453 nm) and emission maxima (475 nm and 501 nm) are similar to other cyan emission variants.^35^–^37^

### Transfections

Transient transfections were used to introduce pECFP-Golgi (Clontech Laboratories Inc., Palo Alto, CA, USA) and the recombinant vectors carrying GFP-GLUT1 fusion proteins into the cells. Liposome-mediated transfection using LipoFectAmine Plus Reagent (#10964013, GibcoBRL, Life Technologies Inc., Rockville, MD, USA) was performed in 35-mm dishes, containing 5 × 10^5^ cells per plate (60%–80% confluent). Each plate was transfected with 1 μg of the vector according to the manufacturer’s instructions. Transient transfections were checked for fluorescent signal at 48–72 hours, when maximal expression of the fluorescent signal was noted in 20%–30% of the cells, based on previous experiments (data not shown). Those transfected with pECFP-Golgi were then fixed for immunocytochemistry.

### Immunocytochemistry

Cells were grown on glass coverslips and fixed. Cells were permeabilized with 0.1% Triton X-100 in PBS. Treatment with primary antibody in 0.1% horse serum in PBS was performed overnight at 4°C.

For immunocytochemistry, cells were treated with the following primary antibodies: (1) A highly specific, non-species-specific, peptide affinity purified mouse polyclonal antibody against a portion of the C-terminus of GLUT1, 6 μg/mL;^38^ (2) A mouse monoclonal antibody against human α-lactalbumin, the milk whey protein that associates with galactosyltransferase to form lactose synthetase (Clone F20.16) (NeoMarkers, Lab Vision Corp., Fremont, CA, USA), 1:100;^10^,^39^–^41^ (3) A mouse monoclonal antibody against rat mannosidase II, a medial-Golgi marker (Clone 53FC3) (BabCO, Berkeley Antibody Company, Richmond, CA, USA), 1:100;^42^–^44^ and (4) A mouse monoclonal antibody against synthetic peptide D1 of β-COP, a *cis*-Golgi marker that also marks the *trans-*Golgi network and the ER-Golgi intermediate compartment (Clone maD) (Sigma, St. Louis, MO, USA), 1:80.^45^–^47^

FITC-conjugated goat anti-rabbit antibody and Texas Red-conjugated rabbit anti-mouse antibody (diluted 1:100 with 0.1% horse serum in PBS) were used as secondary antibodies. Coverslips were then mounted in Pro Long anti-fade medium (Molecular Probes, Eugene, OR, USA) on glass slides for microscopic examination.

### Cell Staining

#### Staining with BODIPY-TR Ceramide

For staining the *trans*-Golgi subcompartment, cells were stained with BODIPY-TR ceramide.^48^–^50^ Cells were grown on glass coverslips and fixed. Incubation with 5 nmol/mL BODIPY-TR ceramide (Molecular Probes, Inc., Eugene, OR, USA) was performed on permeabilized cells in the same manner used for the treatment with the primary antibodies, as described above.

#### Staining with Transferrin-Texas Red

A brief exposure to transferrin-Texas Red served to mark endosomes.^51^,^52^ Cells were grown on glass coverslips, and incubated with 100 μg/mL of transferrin-Texas Red (Molecular Probes, Inc., Eugene, OR, USA) at 37°C for 15 minutes to mark endosomes, before fixation, permeabilization, and staining with anti-GLUT1 antibody.

### Fluorescent Microscopy

Fluorescent signal was detected using an OLYMPUS iX-70 epifluorescent microscope. Images were captured by an uncooled color CCD camera (Optronics, DEI-750 CE Digital Output Model S60675). Exposure was adjusted in a linear manner, and separate color channels were merged as indicated using Adobe Photoshop 5.0 software. For the study of changes taking place in cells over time and under different conditions, cells were grown on round coverslips, and were maintained in a 37°C chamber. Images were acquired sequentially to avoid cross-over. Time-lapse images were captured every 1–5 minutes (depending on the rate of changes) using Snappy software. After linear adjustments of the images in Photoshop 5.0, as described above, the time-lapse images were combined into a sequence using Macromedia Flash 4.0 software. Each condition was studied at least three times and at least on five or six different cells. The results were consistent in all cases. Representative results are shown.

### Hormones

CIT3 cells transfected with GLUT1-EGFP and kept in GM were exposed to SM. Cells in GM were afterwards exposed to SM without hydrocortisone, containing different concentrations of prolactin (3 μg/mL as in full SM, as well as lower concentrations of 300, 30, and 3 ng/mL). To exclude the possibility of a hydrocortisone effect, cells in GM were exposed to SM containing hydrocortisone (3 μg/mL, as in full SM), but no prolactin. Time-lapse images of the changes taking place in GLUT1-EGFP fluorescent signal subcellular targeting were recorded.

## RESULTS

### GLUT1 Subcellular Targeting in HMEC

**Figure 1:**
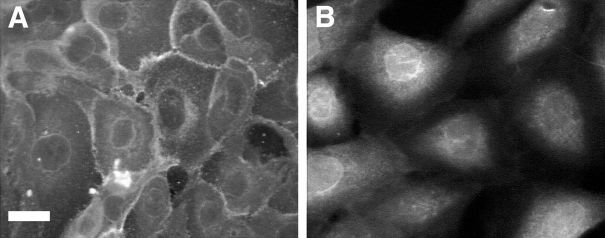
Exposure to Prolactin Causes Intracellular Targeting of GLUT1 Cells were fixed and exposed to specific anti-GLUT1 primary antibody. Bar 15 μm. **A:** In maintenance medium, GLUT1 demonstrates primarily a plasma membrane distribution as well as some intracellular mostly perinuclear staining. **B:** After exposure to prolactin-rich medium for 4 days, GLUT1 was specifically targeted intracellularly, demonstrating a perinuclear pattern, as well as a distinct nuclear membrane staining.

Immunocytochemistry of HMEC in maintenance MEGM medium using highly specific anti-GLUT1 antibody demonstrated plasma membrane distribution of GLUT1 as well as an intracellular, mostly perinuclear, pattern (Figure 1A). After exposure to the prolactin-rich MESM for 4 days, GLUT1 was specifically targeted intracellularly, demonstrating a perinuclear pattern. A distinct nuclear membrane distribution of GLUT1 was also observed under these conditions (Figure 1B).

**Figure 2:**
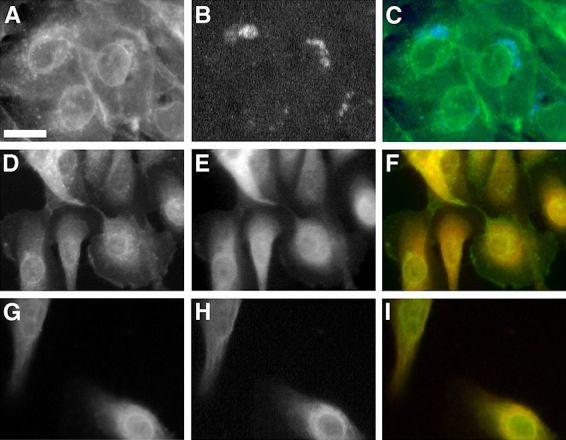
After Exposure to Prolactin, GLUT1 Co-localizes with ECFP-Golgi, α-Lactalbumin and α-Mannosidase II Fluorescent images were captured 60 hours after transfection with 1 μg of pECFP-Golgi. Cells were maintained in prolactin-rich medium for 4 days, before they were fixed and stained with specific anti-GLUT1, anti-α-lactalbumin or anti-α-mannosidase II. GLUT1 is shown in green, and α-lactalbumin or α-mannosidase II in red after staining with FITC-conjugated and Texas Red-conjugated secondary antibodies, respectively. ECFP-Golgi emits cyan-blue fluorescence when exposed to fluorescent light at the appropriate wavelength. Bar 10 μm. **A, D, G:** GLUT1 signal. **B:** ECFP-Golgi signal. **E, H:** α-Lactalbumin and α-mannosidase II signals, respectively. **C, F, I:** Superimposed images. Perinuclear co-localization of GLUT1 and ECFP-Golgi is shown as areas of coincident staining **(C)**. Co-localization of GLUT1 and α-lactalbumin or α-mannosidase II appear as areas of coincident staining, giving rise to yellow signal **(F, I)**.

**Figure 3:**
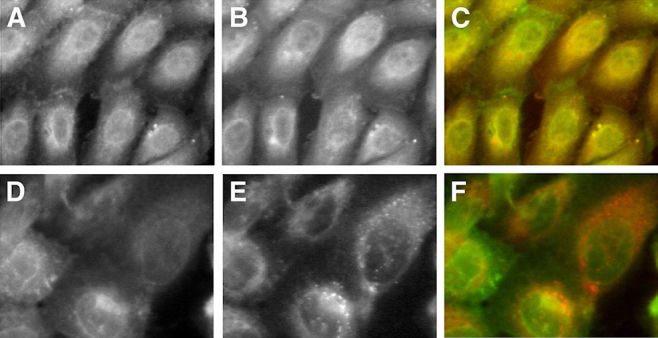
After Exposure to Prolactin, GLUT1 only Partially Co-localizes with β-COP and Transferrin-Texas Red in Endosomes Cells were maintained in prolactin-rich medium for 4 days, before they were fixed and stained with specific anti-GLUT1 or anti-β-COP primary antibodies. Some cells were exposed shortly to transferrin-Texas Red staining before fixation and exposure to anti-GLUT1. GLUT1 is shown in green, and β-COP in red after staining with FITC-conjugated and Texas Red-conjugated secondary antibodies, respectively. Transferrin stain appears in red. **A, D:** GLUT1 signal. **B, E:** β-COP and transferrin (short-term exposure) signals, respectively. **C, F:** Superimposed images. Partial co-localization of GLUT1 with β-COP or transferrin in endosomes appears as areas of coincident staining, giving rise to yellow signal.

**Figure 4:**
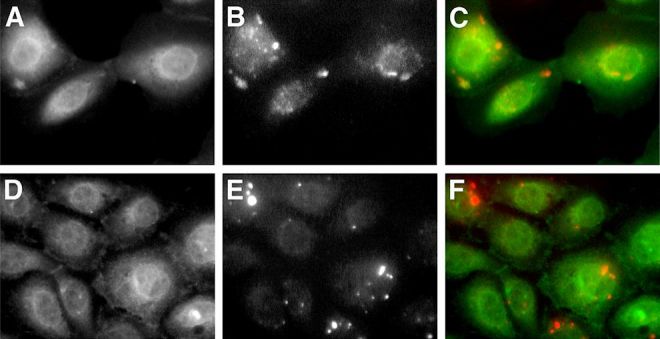
After Exposure to Prolactin, GLUT1 Does not Co-localize with BODIPY-TR Ceramide Cells were maintained in prolactin-rich medium for 4 days, before they were fixed and stained with specific anti-GLUT1 primary antibody, or stained with BODIPY-TR ceramide. GLUT1 is shown in green after staining with FITC-conjugated secondary antibody. BODIPY-TR ceramide appears in red. **A, D:** GLUT1 signal. **B, E:** BODIPY-TR ceramide signal. **C, F:** Superimposed image. There is little overlap of GLUT1 green signal with BODIPY-TR ceramide.

In secretion medium GLUT1 green signal co-localized with the blue signal of pECFP-Golgi (Figure 2A, B, C). It also co-localized with the red signal of α-lactalbumin and α-mannosidase II (Figure 2D, E, F, and G, H, I, respectively). Partial co-localization was demonstrated with the red signal of β-COP and with transferrin-Texas Red (Figure 3A, B, C, and D, E, F, respectively). No co-localization was demonstrated after staining the cells with the red stain, BODIPY-TR ceramide (Figure 4A, B, C, D, E, F).

### GLUT1 Fusion Chimeras to EGFP Exhibit Normal GLUT1 Targeting *in Vitro*

We subcloned GLUT1 cDNA into pEGFP to create N- and C-terminus fusions. The recombinant plasmid vectors were introduced into CIT3 cells by transient liposome-mediated transfection, achieving fluorescent expression in 20%–30% of the cells.

**Figure 5:**
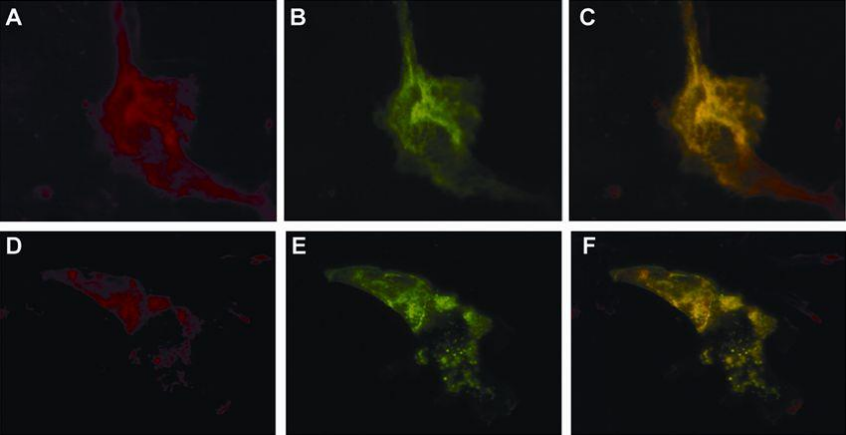
GLUT1 Chimeras to GFP Co-localize with Native GLUT1 in CIT3 Cells in SM EGFP-GLUT1 fusion protein **(B)** exhibits the same intracellular distribution as native GLUT1 **(A)**. Superimposed images **(C)** demonstrate that co-localization of native GLUT1 and EGFP-GLUT1 fusion protein appears as areas of coincident staining, giving rise to yellow signal. GLUT1-EGFP fusion protein **(E)** exhibits the same intracellular distribution as native GLUT1 **(D)**. Superimposed images **(F)** demonstrate that co-localization of native GLUT1 and GLUT1-EGFP fusion protein appears as areas of coincident staining, giving rise to yellow signal.

Immunocytochemistry studies with antibodies against the C-terminus of native GLUT1 showed that the fluorescent signal of both GLUT1 chimeras to GFP co-localized extensively with native GLUT1 (Figure 5). This result validated the use of the GLUT1-GFP fusion proteins to study dynamic aspects of GLUT1 targeting.

**Figure 6:**
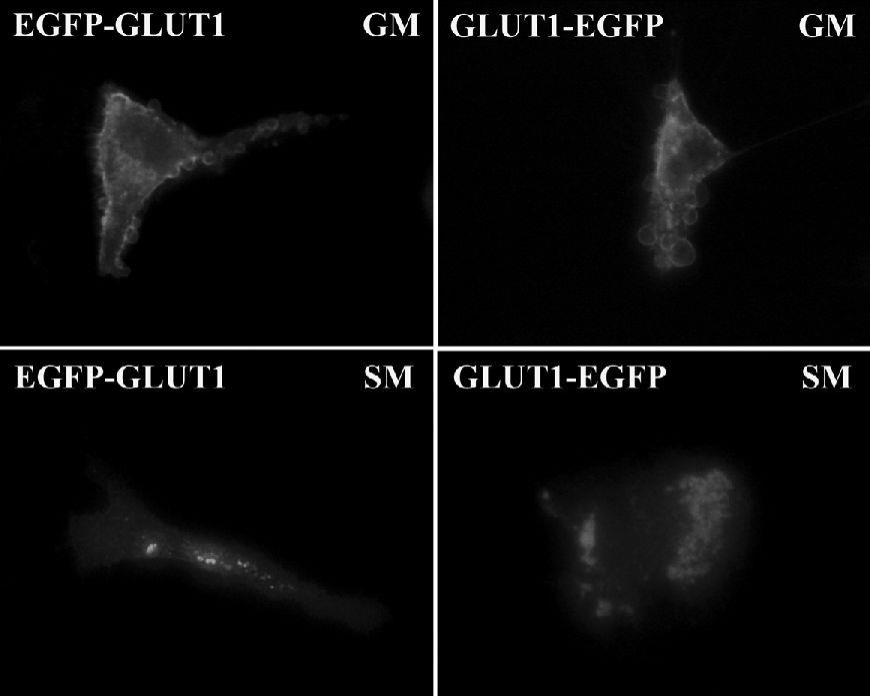
CIT3 Cells Expressing EGFP Fusion to the N- and C-termini of GLUT1 in GM and after 4 Days in SM High-power images. **Upper left:** Plasma membrane targeting of EGFP-GLUT1 in GM. **Lower left:** Intracellular pattern of EGFP-GLUT1 signal in differentiated cells in SM. **Upper right:** Plasma membrane targeting of GLUT1-EGFP in GM. **Lower right:** Intracellular targeting of GLUT1-EGFP with perinuclear punctate distribution in cells exposed to SM. All images captured with 0.25 s exposure time and are at 40× magnification, except for the upper left image, which was optimized at 0.5 s exposure time and higher power (60×).

EGFP chimeras to GLUT1 demonstrated change in subcellular distribution, after 96 hours of exposure to SM. In GM the fusion proteins were targeted mainly to the basolateral plasma membrane. In SM GLUT1-EGFP chimeras were mostly targeted into the cell, exhibiting unique perinuclear distribution with punctate pattern scattered through the cytoplasm (Figures 5 and 6). Both the N- and C-terminus fusions to GLUT1 (EGFP-GLUT1 and GLUT1-EGFP, respectively) exhibited the same targeting patterns (Figure 6).

### Degree of Differentiation Affects Intracellular Targeting of GLUT1

**Figure 7:**
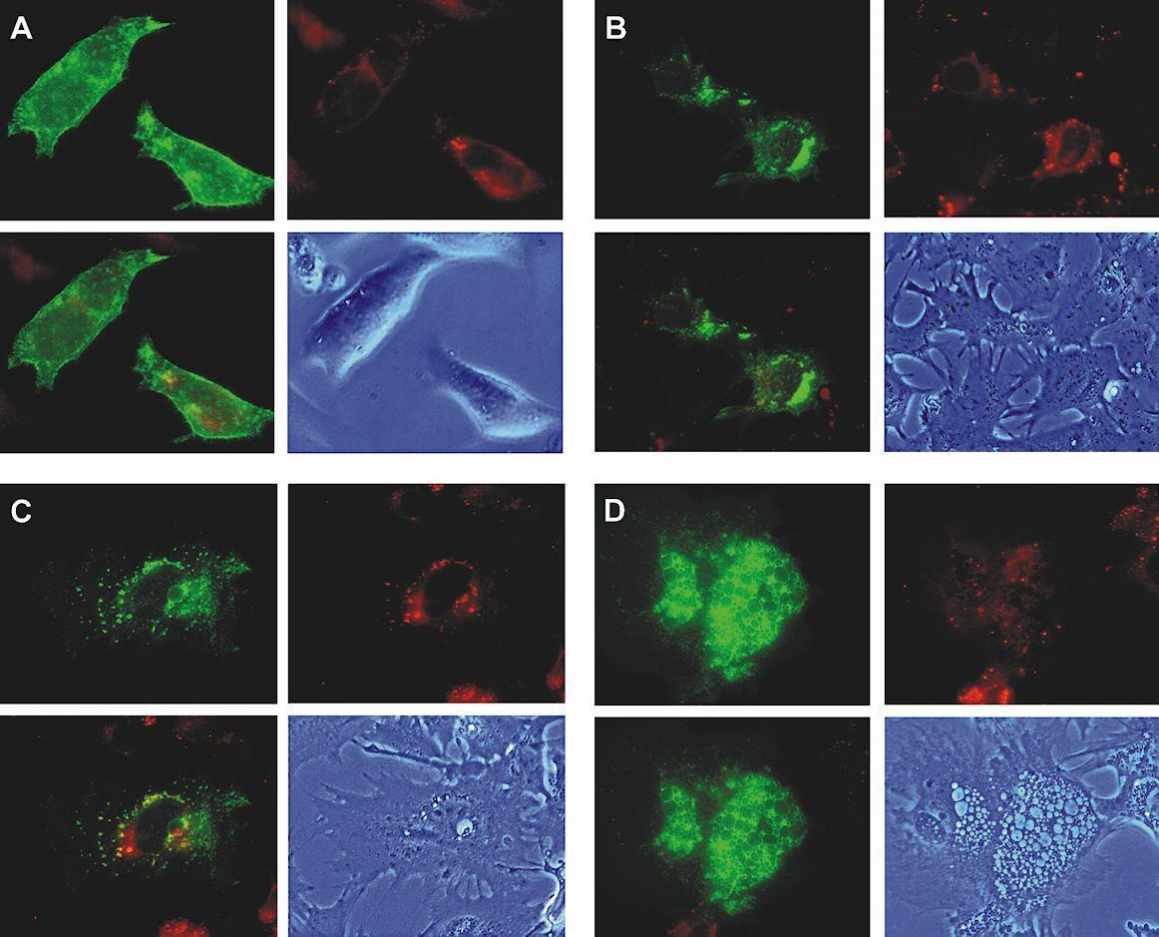
Degree of Differentiation Affects Intracellular Targeting of GLUT1 Static images of CIT3 cells in GM and at different levels of differentiation in SM. Panel **A:** In GM; **B, C, D:** In SM at different levels of differentiation. The upper left figure of each panel is GLUT1-EGFP signal. The upper right figure is staining of the same cells with BODIPY-TR ceramide (Molecular Probes, Inc., Eugene, OR, USA), which is a dye that marks the *trans*-Golgi in red. Living CIT3 cells were pre-incubated with 5 nmol/mL of BODIPY-TR ceramide at 4°C for 30 minutes. Lower left figure is superimposed image of the upper figures. Lower right figure is phase contrast image of the cells to show the different levels of differentiation.

Static images of cells at different levels of differentiation in SM demonstrated that the degree of differentiation affected the level and distribution of GLUT1 targeting in perinuclear localization (Figure 7). In GM the GLUT1-EGFP signal was mostly targeted to the plasma membrane (Figure 7A). In SM GLUT1-EGFP fusion proteins exhibited the unique perinuclear punctate pattern, described above (Figure 7B). However, in the more differentiated MEC in SM, where vesicles and fat globules were morphologically prominent, the GLUT1-EGFP signal was no longer punctate, but targeted to the boundaries of these vesicles, still maintaining mostly perinuclear distribution. There was signal throughout the cytoplasm as well (Figure 7C, D). This differentiation-related GLUT1 subcellular targeting may also support the assumption that dynamic transport systems are involved in the trafficking of the GFP-GLUT1 fusion proteins, as will be shown below.

### GLUT1 Intracellular Trafficking in Secretion Media Seems to be Dynamic

**Figure 8:**
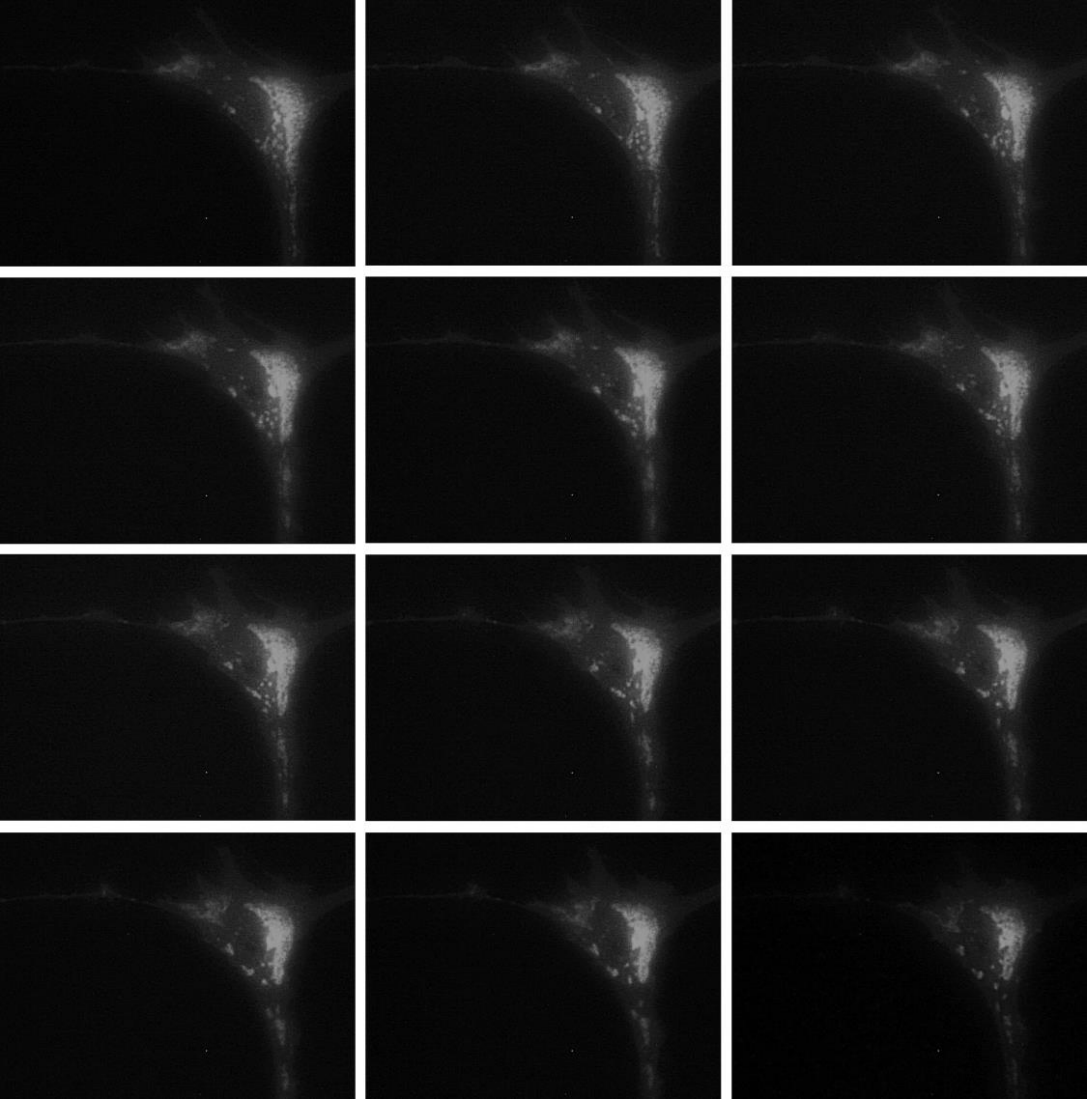
GLUT1 Intracellular Trafficking in SM is Dynamic Figure 8 is also available online as a YouTube video clip at: https://youtu.be/lEby2cqSDek. The figure plates are from frames 8 minutes apart. All images captured with 0.25 s exposure time and are at 40× magnification.

Living CIT3 cells transfected with GLUT1-EGFP and kept in SM were followed by time-lapse imaging, where trafficking of GLUT1 fusion proteins could be seen (Figure 8; also available online as a supplemental YouTube video clip). This trafficking seems to be dynamic and could suggest that transport systems are possibly involved in it.

### Secretion Medium Induces GLUT1 Intracellular Targeting in Living Mammary Cells

**Figure 9:**
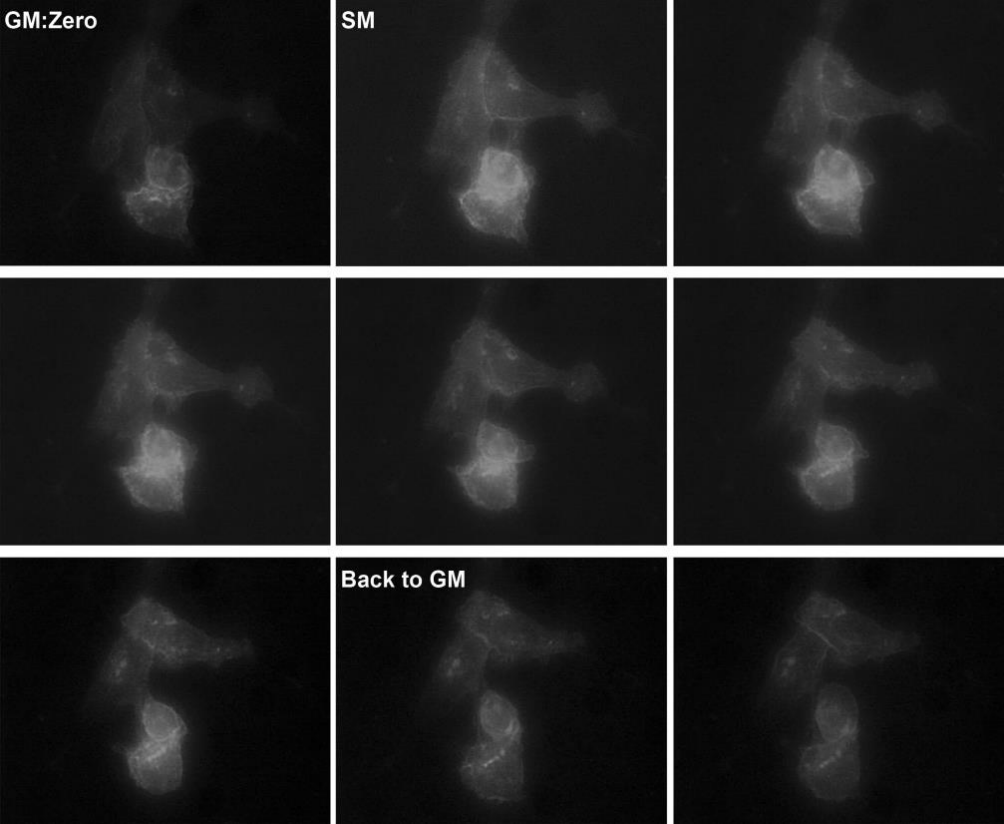
Lactogenic Hormones in SM Induce GLUT1 Intracellular Targeting in Living Mammary Cells Figure 9 is also available online as a YouTube video clip at: https://youtu.be/3oYv21jcAj4. The figure plates are from frames 16 minutes apart. All images captured with 0.5 s exposure time and are at 40× magnification.

When living CIT3 cells, transfected with GLUT1-EGFP and kept in GM, were exposed to SM, dynamic trafficking of GLUT1 fusion proteins was demonstrated intracellularly, starting after approximately 50–60 minutes, with maximal intracellular targeting within 90–110 minutes. When the cells were returned to GM, most of the changes were reversible within 1–2 hours, although not fully, with redistribution of the fluorescent GLUT1 chimera mostly in the plasma membrane (Figure 9; also available online as a supplemental YouTube video clip).

### Prolactin Induces GLUT1 Intracellular Targeting in Living Mammary Cells

**Figure 10:**
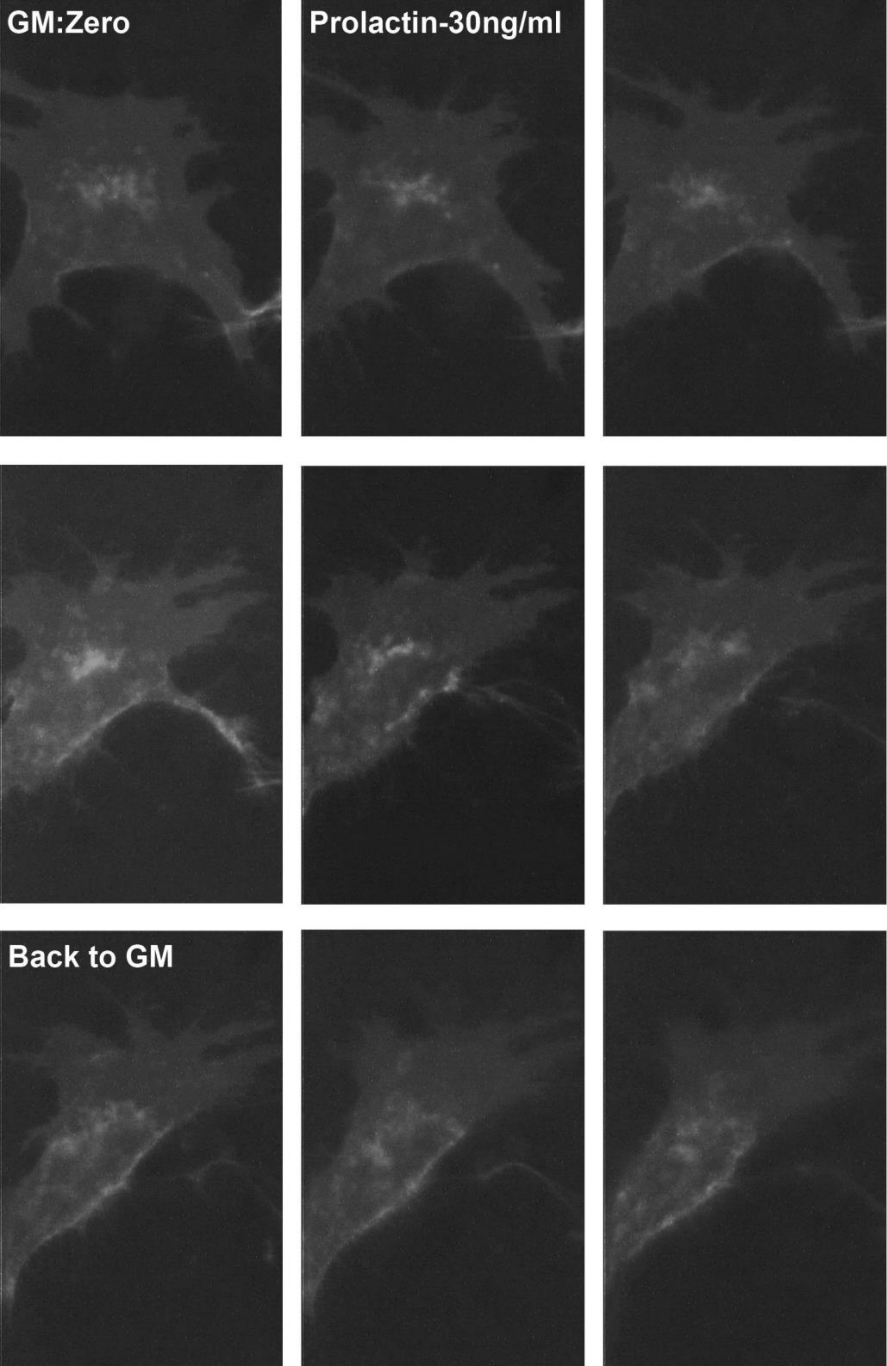
Prolactin Induces GLUT1 Intracellular Targeting in Living Mammary Cells The response here was reproduced with prolactin concentrations of 30 ng/mL. Figure 10 is also available online as a YouTube video clip at: https://youtu.be/bUkUHZ0soPI. The figure plates are from frames 20 minutes apart. All images captured with 2 s exposure time and are at 60× magnification.

Exposure of CIT3 cells kept in GM to SM containing prolactin without hydrocortisone caused the same changes in GLUT1 subcellular targeting as were seen with full SM. Dynamic trafficking of GLUT1 fusion proteins intracellularly was demonstrated, starting after approximately 50–60 minutes, with maximal intracellular targeting within 90–110 minutes. When the cells were returned to GM, most of the changes were reversible within 1–2 hours, although not fully. The same response was reproduced with different prolactin concentrations (300 ng/mL, 30 ng/mL), as low as 3 ng/mL (compared to the 3 μg/mL of prolactin usually used in SM). Representative results after exposure of CIT3 cells kept in GM to 30 ng/mL prolactin are shown (Figure 10; also available online as a supplemental YouTube video clip). We were not able to demonstrate dose-response relations with the different prolactin concentrations, possibly because the results are qualitative, rather than quantitative. There was no difference in the time required to achieve maximal effect with different prolactin concentrations.

Secretion medium containing hydrocortisone (3 μg/mL as in full SM) without any prolactin caused no change in GLUT1 subcellular distribution.

## DISCUSSION

Human milk contains about three times more lactose than does rodent milk, suggesting that glucose transport mechanisms can be very important in humans. Thus, before studying GLUT1 subcellular trafficking in a CIT3 MMEC model, we first examined whether hormonally regulated subcellular targeting of GLUT1 occurs in HMEC upon conditions mimicking lactation. For this, we utilized immunofluorescent staining of HMEC to demonstrate prolactin-dependent co-localization of GLUT1 with several Golgi markers.

The distribution of GLUT1, as demonstrated using immunocytochemistry with C-terminus-specific anti-GLUT1 antibody, was primarily in plasma membrane, as well as some intracellular perinuclear punctate pattern, when the cells were maintained in baseline growth medium. Upon exposure to prolactin in secretion media, GLUT1 specifically redistributed from the plasma membrane, demonstrating a perinuclear distribution with a pattern that may represent the Golgi or related structures in the central intracellular vacuolar trafficking system. A distinct perinuclear membrane distribution of GLUT1 was also observed under these conditions.

Co-localization studies gave insight as to the possible Golgi subcompartment that GLUT1 resides in. GLUT1 was targeted intracellularly, co-localizing with components of lactose synthetase complex. It co-localized with ECFP-Golgi, the cyan fluorescent protein fused to the membrane-anchoring signal specific to β1,4-galactosyltransferase, identifying the medial/trans region of the Golgi. This co-localization gave another spatial support to its role in the transport of free glucose for lactose synthesis by β1,4-galactosyltransferase in the inner cisternae of the Golgi apparatus. This was further supported by the co-localization of GLUT1 with α-lactalbumin, which is the milk whey protein that is not catalytically active, by itself, but which in association with β1,4-galactosyltransferase is necessary for the synthesis of lactose.^53^ The initiation of α-lactalbumin synthesis that occurs at parturition is required for the initiation of copious milk production, but is neither the only factor nor the limiting factor controlling lactose synthesis.^54^ GLUT1 also co-localized with the medial-Golgi marker, α-mannosidase II; however, it did not co-localize with the *trans*-Golgi marker, BODIPY-TR ceramide. Only partial co-localization was demonstrated with β-COP, which is a *cis*-Golgi marker.

The co-localization studies suggest intracellular targeting to the central vesicular transport system, which may represent a *cis/medial-*Golgi subcompartment. These findings are in agreement with previous findings in the mouse mammary gland^16^ and in CIT3 MMEC.^25^,^55^ Partial co-localization was also noted with transferrin-Texas Red after brief exposure, which marks the endosomes. The possible identification of GLUT1 in endosomes suggests that GLUT1 sorting is a continuous, dynamic process.^55^ Further work delineating the molecular mechanism of GLUT1 sorting and the targeting determinants it recognizes should improve our understanding of a key regulatory step of milk production in the nursing mother.

Apparent nuclear membrane staining for GLUT1 seen in HMEC has not been reported previously in any cell type, and its significance is a matter for speculation and further study. There is evidence to suggest that the apical nuclear envelope may serve as an intermediary connection between the endoplasmic reticulum and the Golgi.^56^ Also, the perinuclear staining of GLUT1 noted may actually be one of the perinuclear endosomal recycling compartments.^52^

Our findings in HMEC illustrate a potential mechanism for the delivery of free glucose to the Golgi, in what may be the rate-limiting step for lactose synthesis and milk production. In addition to its widely recognized role in the uptake of glucose by cells, GLUT1 may also mediate glucose transport between intracellular compartments.

The *in vitro* study of a dynamic process required developing a system of living cells with labeling of GLUT1. Green fluorescent protein is a reporter molecule for monitoring gene expression and protein localization *in vivo*, *in situ*, and in real time.^57^–^63^ Green fluorescent protein is expressed in eukaryotic cells as a fusion protein that serves as a “fluorescent tag.” The use of fluorescent fusion proteins of GLUT1 allows the study of the same cells over time, permitting studies of exocytosis and endocytosis, not just steady-state distributions. Using GFP fusion to GLUT1 is ideal for studying intracellular trafficking and subcellular targeting of GLUT1 in MEC under hormonal stimulation. It also permits evaluation of chimeric protein targeting in an antibody-independent fashion and confirms that we are studying GLUT1 and not a novel, lactation-specific glucose transporter isoform that shares the GLUT1 epitope. The GLUT1 cDNA sequence^29^ was subcloned into pEGFP and introduced into CIT3 cells by transient transfection, with over-expression of the fluorescent GLUT1 in approximately a quarter of the cells. The intracellular targeting of GLUT1-EGFP chimera was consistent from cell to cell.

Lactogenic hormones in SM changed subcellular targeting of GFP fusion chimeras to GLUT1 from a plasma membrane distribution to an intracellular pattern, predominantly perinuclear and punctate, but also throughout the cytoplasm. The fact that this pattern was consistent with the distribution of native GLUT1 supported the use of GLUT1 chimeras to GFP as a model for studying GLUT1 intracellular targeting in MEC. Since the behavior and intracellular distribution of both the N- and C-fusion chimeras of GLUT1 to GFP were consistently the same, further studies were carried out with only one of them (GLUT1-EGFP).

The level of differentiation of the lactating MEC in SM affected the degree of GLUT1 intracellular targeting and the distribution of its cytoplasmic, mostly perinuclear, localization. This fits well the previous *in vivo* and *in vitro* findings^16^,^25^ and actually also points to the possible involvement of intracellular membrane vesicular trafficking systems in GLUT1 intracellular targeting.

Living mouse MEC kept in SM demonstrated dynamic trafficking of GLUT1-EGFP fusion proteins. Careful tracking of these fluorescent GLUT1 vesicles excluded random movement and actually suggested that the dynamic intracellular targeting of GLUT1 may be mediated through altering GLUT1 exocytosis and endocytosis.

When CIT3 cells kept in GM were exposed to SM, the changes in GLUT1 targeting from mostly a plasma membrane pattern to an intracellular pattern occurred within 60–120 minutes. The maximal intracellular translocation of GLUT1-EGFP green fluorescent signal after exposure to SM was noted at 100–110 minutes. Some of this effect was reversible within 60–120 minutes upon withdrawal of SM, but we were not able to demonstrate full reversibility of the process in our *in vitro* system. The relatively rapid changes in GLUT1 targeting in living MMEC exposed to SM, which took place within minutes to hours, were in accordance with the findings from the *in vivo* studies of forced weaning, demonstrating reversible changes in GLUT1 subcellular targeting within 3–5 hours.^16^ These findings are also supported by the previous observation that, as early as 15 minutes after exposure of mammary tissue fragments from lactating rabbits to prolactin, the cell morphology already changed with marked increase in the relative volume occupied by the Golgi region.^64^

The next step was to define which of the hormones in SM is responsible for GLUT1 intracellular targeting. Exposure of CIT3 cells kept in GM to SM containing prolactin without hydrocortisone caused the same changes in GLUT1 subcellular targeting as seen with full SM. The same response was reproduced with prolactin concentrations as low as 3 ng/mL (compared to the 3 μg/mL of prolactin usually used in SM). The serum concentration range of prolactin in lactating mothers is 20–300 ng/mL.^65^ However, we were not able to demonstrate dose-response effects with the different prolactin concentrations. The GLUT1-EGFP intracellular signal translocation took place at approximately the same time (100–120 minutes) with 3 ng/mL prolactin as it did with 3 μg/mL of prolactin in the full SM. Further studies are needed to demonstrate dose-dependent effects of prolactin, expressed as different levels of intracellular green fluorescent signal of GLUT1 chimeras, but this requires a more quantitative recording of the signal that unfortunately we did not have in these studies. Secretion medium containing hydrocortisone without any prolactin caused no change in GLUT1 subcellular distribution, thus excluding it as a cause for GLUT1 intracellular targeting in SM. Further studies are also needed to explore the effects of prolactin combined with other hormones, such as estrogen.

The suggestion that GLUT1 does not solely act at the plasma membrane, but may function in an intracellular organelle as well, conceptually complements the well-known insulin-regulated targeting of GLUT4,^66^ and to a lesser extent of GLUT1,^38^ to their site of action, the plasma membrane, in fat and muscle cells. Our results suggest the existence of a prolactin-induced, cell type-specific, developmental stage-specific sorting machinery for GLUT1 in MEC, and supports glucose transport as a potential rate-limiting step for lactose synthesis during lactation. The ability of the system to respond quickly to hormonal changes by altering the transport, and thus the availability of free glucose for lactose synthesis, is complementary to the well-known insulin-regulated targeting of GLUT4 to the plasma membrane in fat and muscle cells, where GLUT4 is available for glucose uptake into the cell within minutes.^67^ This machinery offers another level of regulation of lactose synthesis by altering GLUT1 targeting within minutes to hours, as was demonstrated also *in vivo*.^16^ This step may not require new protein synthesis, or increase in the total amount of GLUT1 or enzymes involved in lactose synthesis, which takes longer. Our study relied only on immuno-histochemistry analysis, and further studies including Western blot and real-time PCR are needed to address the possibility of up- or down-regulation of GLUT1.

Despite the fact that in our first experiments we have demonstrated specific intracellular targeting of GLUT1 from the plasma membrane of HMEC upon exposure to prolactin, the main limitation of this part of the dynamic studies is that it was limited to MMEC, and more specifically to CIT3 cell lines. Thus, these conclusions cannot be currently generalized or extended to other mammals, including humans. The suggestion that glucose transporters, other than GLUT1, may be involved in glucose regulation in MEC during lactation,^18^,^68^–^71^ and that their role may be more significant in other mammals, and influenced by factors other than lactogenic hormones,^72^–^75^ needs to be addressed.

Another limitation of this study is the issue of cell-to-cell variability in the phenotype of mouse MEC in the culture. Cell phenotype could vary in many ways, including morphology, intracellular lipid deposition, apparent states of differentiation, and GLUT1 targeting kinetics. This is an inherent limitation of a descriptive study based on microscopic findings. To decrease a possible selection bias of the cells we were studying, we tried to select representative cells on a lower-power field before studying them in a high-power field. We also repeated each experiment more than three times, and verified that the results were reproducible. Yet, such selection bias cannot be fully excluded.

A further limitation of our descriptive co-localization studies is the use of fluorescent microscopy. Confocal microscopy should be preferred in future studies like this, because it gives higher resolution and better color separation, enables the use of more colors at the same time, and is more accurate in differentiating true co-localization from proteins in close proximity.

Also, being a descriptive morphological study, our study did not deal with the ability of the cells to synthesize and secrete lactose in culture. This is a complex issue that is not dependent only on the presence of lactogenic hormones in the medium, and may be affected by many factors, such as the intracellular matrix. However, if these results were obtained in cells expressing lactose, then the issues of cell-to-cell variations and possible phenotypic effects discussed above would have been minimized.

The fact that this work is based on two previous works, one *in vivo* on mammary glands from lactating mice^16^ and the second *in vitro* on the same CIT3 cells,^25^,^55^ establishes a continuum that supports our results within their limited scope. These results can possibly form the basis and methodological approach for future works in other primary MEC in order to try and generalize the conclusions.

In summary, our results demonstrated a basal constitutive GLUT1 membrane-recycling pathway between an intracellular pool and the cell surface in CIT3 MMEC, which targets most of the GLUT1 to the plasma membrane in GM. As in other cell types it is responsible for maintaining basal glucose uptake. But, in these MEC there is hormonally regulated cell type-specific, developmental stage-specific sorting machinery for GLUT1 intracellular targeting in lactation. This process is induced by prolactin and is highly sensitive to low concentrations of prolactin. It provides the cell with a quick mechanism by which it can supply free glucose intracellularly to serve as substrate for lactose synthesis in the Golgi. This machinery offers another level of regulation of lactose synthesis by altering GLUT1 targeting within minutes to hours, as was demonstrated also *in vivo.*^16^ The rapid responsiveness of GLUT1 targeting suggests that this machinery does not require new protein synthesis. It may also support glucose transport as a rate-limiting step for lactose synthesis during lactation.

## Abbreviations

ECFP: enhanced cyan fluorescent protein
EGFP: enhanced GFP
GFP: green fluorescent protein
GM: growth medium
HMEC: human mammary epithelial cells
MEC: mammary epithelial cells
MMEC: mouse mammary epithelial cells
pECFP: plasmid vector ECFP
pEGFP: plasmid vector EGFP
PCR: polymerase chain reaction
SM: secretion medium.

## References

[1] Oftedal OT (2012). The evolution of milk secretion and its ancient origins. Animal.

[2] Capuco AV, Akers RM (2009). The origin and evolution of lactation. J Biol.

[3] Lefevre CM, Sharp JA, Nicholas KR (2010). Evolution of lactation: ancient origin and extreme adaptations of the lactation system. Annu Rev Genomics Hum Genet.

[4] Dall SR, Boyd IL (2004). Evolution of mammals: lactation helps mothers to cope with unreliable food supplies. Proc Biol Sci.

[5] Skibiel AL, Downing LM, Orr TJ, Hood WR (2013). The evolution of the nutrient composition of mammalian milks. J Anim Ecol.

[6] McManaman JL, Neville MC (2003). Mammary physiology and milk secretion. Adv Drug Deliv Rev.

[7] Svennersten-Sjaunja K, Olsson K (2005). Endocrinology of milk production. Domest Anim Endocrinol.

[8] Strange R, Metcalfe T, Thackray L, Dang M (2001). Apoptosis in normal and neoplastic mammary gland development. Microsc Res Tech.

[9] Zhao FQ (2014). Biology of glucose transport in the mammary gland. J Mammary Gland Biol Neoplasia.

[10] Strous GJ (1986). Golgi and secreted galactosyltransferase. CRC Crit Rev Biochem.

[11] Dils RR (1989). Synthetic and secretory processes of lactation. Proc Nutr Soc.

[12] Nicholas KR, Hartmann PE, McDonald BL (1981). Alpha-Lactalbumin and lactose concentrations in rat milk during lactation. Biochem J.

[13] Joost HG, Thorens B (2001). The extended GLUT-family of sugar/polyol transport facilitators: nomenclature, sequence characteristics, and potential function of its novel members (review). Mol Membr Biol.

[14] Burnol AF, Leturque A, Loizeau M, Postic C, Girard J (1990). Glucose transporter expression in rat mammary gland. Biochem J.

[15] Camps M, Vilaro S, Testar X, Palacin M, Zorzano A (1994). High and polarized expression of GLUT1 glucose transporters in epithelial cells from mammary gland: acute down-regulation of GLUT1 carriers by weaning. Endocrinology.

[16] Nemeth BA, Tsang SW, Geske RS, Haney PM (2000). Golgi targeting of the GLUT1 glucose transporter in lactating mouse mammary gland. Pediatr Res.

[17] Shennan DB (1998). Mammary gland membrane transport systems. J Mammary Gland Biol Neoplasia.

[18] Macheda ML, Williams ED, Best JD, Wlodek ME, Rogers S (2003). Expression and localisation of GLUT1 and GLUT12 glucose transporters in the pregnant and lactating rat mammary gland. Cell Tissue Res.

[19] Zhao FQ, Dixon WT, Kennelly JJ (1996). Localization and gene expression of glucose transporters in bovine mammary gland. Comp Biochem Physiol B Biochem Mol Biol.

[20] Zhao FQ, Moseley WM, Tucker HA, Kennelly JJ (1996). Regulation of glucose transporter gene expression in mammary gland, muscle, and fat of lactating cows by administration of bovine growth hormone and bovine growth hormone-releasing factor. J Anim Sci.

[21] Hudson ER, Ma LS, Wilde CJ, Flint DJ, Baldwin SA (1997). Regulation of GLUT1 expression in the mammary gland. Biochem Soc Trans.

[22] Harris DS, Slot JW, Geuze HJ, James DE (1992). Polarized distribution of glucose transporter isoforms in Caco-2 cells. Proc Natl Acad Sci U S A.

[23] Madon RJ, Martin S, Davies A, Fawcett HA, Flint DJ, Baldwin SA (1990). Identification and characterization of glucose transport proteins in plasma membrane- and Golgi vesicle-enriched fractions prepared from lactating rat mammary gland. Biochem J.

[24] Takata K, Fujikura K, Suzuki M, Suzuki T, Hirano H (1997). GLUT1 glucose transporter in the lactating mammary gland in the rat. Acta Histochem Cytochem.

[25] Haney PM (2001). Localization of the GLUT1 glucose transporter to Brefeldin A-sensitive vesicles of differentiated CIT3 mouse mammary epithelial cells. Cell Biol Int.

[26] Toddywalla VS, Kari FW, Neville MC (1997). Active transport of nitrofurantoin across a mouse mammary epithelial monolayer. J Pharmacol Exp Ther.

[27] Kaether C, Salm T, Glombik M, Almers W, Gerdes HH (1997). Targeting of green fluorescent protein to neuroendocrine secretory granules: a new tool for real time studies of regulated protein secretion. Eur J Cell Biol.

[28] Yang TT, Sinai P, Green G (1998). Improved fluorescence and dual color detection with enhanced blue and green variants of the green fluorescent protein. J Biol Chem.

[29] Mueckler M, Caruso C, Baldwin SA (1985). Sequence and structure of a human glucose transporter. Science.

[30] Watzele G, Bachofner R, Berger EG (1991). Immunocytochemical localization of the Golgi apparatus using protein-specific antibodies to galactosyltransferase. Eur J Cell Biol.

[31] Watzele G, Berger EG (1990). Near identity of HeLa cell galactosyltransferase with the human placental enzyme. Nucleic Acids Res.

[32] Llopis J, McCaffery JM, Miyawaki A, Farquhar MG, Tsien RY (1998). Measurement of cytosolic, mitochondrial, and Golgi pH in single living cells with green fluorescent proteins. Proc Natl Acad Sci U S A.

[33] Yamaguchi N, Fukuda MN (1995). Golgi retention mechanism of beta-1,4-galactosyltransferase. Membrane-spanning domain-dependent homodimerization and association with alpha- and beta-tubulins. J Biol Chem.

[34] Gleeson PA, Teasdale RD, Burke J (1994). Targeting of proteins to the Golgi apparatus. Glycoconj J.

[35] Heim R, Prasher DC, Tsien RY (1994). Wavelength mutations and posttranslational autoxidation of green fluorescent protein. Proc Natl Acad Sci U S A.

[36] Heim R, Tsien RY (1996). Engineering green fluorescent protein for improved brightness, longer wavelengths and fluorescence resonance energy transfer. Curr Biol.

[37] Miyawaki A, Llopis J, Heim R (1997). Fluorescent indicators for Ca2+ based on green fluorescent proteins and calmodulin. Nature.

[38] Gould GW, Derechin V, James DE (1989). Insulin-stimulated translocation of the HepG2/erythrocyte-type glucose transporter expressed in 3T3-L1 adipocytes. J Biol Chem.

[39] Herbert DC, Burke RE, McGuire WL (1978). Casein and alpha-lactalbumin detection in breast cancer cells by immunocytochemistry. Cancer Res.

[40] Wrba F, Reiner A, Ritzinger E, Reiner G, Holzner JH (1986). Immunohistochemistry of alpha-lactalbumin, lactoferrin and trans- ferrin receptor in invasive breast carcinomas with regard to tumor grading, estrogen receptor status and tumor staging. Verh Dtsch Ges Pathol.

[41] Simickova M, Lang BA, Rejthar A, Cernoch M, Sakalova J, Pacovsky Z (1989). Immunohistochemical localization of alpha-lactalbumin in human breast cancer tissue. Neoplasma.

[42] Baron MD, Garoff H (1990). Mannosidase II and the 135-kDa Golgi-specific antigen recognized monoclonal antibody 53FC3 are the same dimeric protein. J Biol Chem.

[43] Burke B, Griffiths G, Reggio H, Louvard D, Warren G (1982). A monoclonal antibody against a 135-K Golgi membrane protein. EMBO J.

[44] Burke B, Warren G (1984). Microinjection of mRNA coding for an anti-Golgi antibody inhibits intracellular transport of a viral membrane protein. Cell.

[45] Griffiths G, Pepperkok R, Locker JK, Kreis TE (1995). Immunocytochemical localization of beta-COP to the ER-Golgi boundary and the TGN. J Cell Sci.

[46] Pepperkok R, Scheel J, Horstmann H, Hauri HP, Griffiths G, Kreis TE (1993). Beta-COP is essential for biosynthetic membrane transport from the endoplasmic reticulum to the Golgi complex in vivo. Cell.

[47] Serafini T, Stenbeck G, Brecht A (1991). A coat subunit of Golgi-derived non-clathrin-coated vesicles with homology to the clathrin-coated vesicle coat protein beta-adaptin. Nature.

[48] Harrington EO, Loffler J, Nelson PR, Kent KC, Simons M, Ware JA (1997). Enhancement of migration by protein kinase Calpha and inhibition of proliferation and cell cycle progression by protein kinase Cdelta in capillary endothelial cells. J Biol Chem.

[49] Pagano RE, Martin OC, Kang HC, Haugland RP (1991). A novel fluorescent ceramide analogue for studying membrane traffic in animal cells: accumulation at the Golgi apparatus results in altered spectral properties of the sphingolipid precursor. J Cell Biol.

[50] Pagano RE, Sepanski MA, Martin OC (1989). Molecular trapping of a fluorescent ceramide analogue at the Golgi apparatus of fixed cells: interaction with endogenous lipids provides a trans-Golgi marker for both light and electron microscopy. J Cell Biol.

[51] Hopkins CR, Gibson A, Shipman M, Miller K (1990). Movement of internalized ligand-receptor complexes along a continuous endosomal reticulum. Nature.

[52] Mayor S, Presley JF, Maxfield FR (1993). Sorting of membrane components from endosomes and subsequent recycling to the cell surface occurs by a bulk flow process. J Cell Biol.

[53] Ramakrishnan B, Shah PS, Qasba PK (2001). alpha-Lactalbumin (LA) stimulates milk beta-1,4-galactosyltransferase I (beta 4Gal-T1) to transfer glucose from UDP-glucose to N-acetylglucosamine. Crystal structure of beta 4Gal-T1 x LA complex with UDP-Glc. J Biol Chem.

[54] Kuhn NJ, Carrick DT, Wilde CJ (1980). Lactose synthesis: the possibilities of regulation. J Dairy Sci.

[55] Riskin A, Nannegari VH, Mond Y (2008). Acute effectors of GLUT1 glucose transporter subcellular targeting in CIT3 mouse mammary epithelial cells. Ped Res.

[56] Hager KM, Striepen B, Tilney LG, Roos DS (1999). The nuclear envelope serves as an intermediary between the ER and Golgi complex in the intracellular parasite Toxoplasma gondii. J Cell Sci.

[57] Chalfie M, Tu Y, Euskirchen G, Ward WW, Prasher DC (1994). Green fluorescent protein as a marker for gene expression. Science.

[58] Dobson SP, Livingstone C, Gould GW, Tavare JM (1996). Dynamics of insulin-stimulated translocation of GLUT4 in single living cells visualised using green fluorescent protein. FEBS Lett.

[59] Drmota T, Gould GW, Milligan G (1998). Real time visualization of agonist-mediated redistribution and internalization of a green fluorescent protein-tagged form of the thyrotropin-releasing hormone receptor. J Biol Chem.

[60] Kain SR, Adams M, Kondepudi A, Yang TT, Ward WW, Kitts P (1995). Green fluorescent protein as a reporter of gene expression and protein localization. Biotechniques.

[61] Lippincott-Schwartz J, Cole N, Presley J (1998). Unravelling Golgi membrane traffic with green fluorescent protein chimeras. Trends Cell Biol.

[62] Presley JF, Cole NB, Schroer TA, Hirschberg K, Zaal KJ, Lippincott-Schwartz J (1997). ER-to-Golgi transport visualized in living cells. Nature.

[63] Tsien RY (1998). The green fluorescent protein. Annu Rev Biochem.

[64] Ollivier-Bousquet M (1978). Early effects of prolactin on lactating rabbit mammary gland. Ultrastructural changes and stimulation of casein secretion. Cell Tissue Res.

[65] Rebar RW, Creasy RK, Resnik R (1999). The Breast and the Physiology of Lactation. Maternal-Fetal Medicine.

[66] Holman GD, Kozka IJ, Clark AE (1990). Cell surface labeling of glucose transporter isoform GLUT4 by bis-mannose photolabel. Correlation with stimulation of glucose transport in rat adipose cells by insulin and phorbol ester. J Biol Chem.

[67] Watson RT, Kanzaki M, Pessin JE (2004). Regulated membrane trafficking of the insulin-responsive glucose transporter 4 in adipocytes. Endocr Rev.

[68] Obermeier S, Huselweh B, Tinel H, Kinne RH, Kunz C (2000). Expression of glucose transporters in lactating human mammary gland epithelial cells. Eur J Nutr.

[69] Zhao FQ, Keating AF (2007). Expression and regulation of glucose transporters in the bovine mammary gland. J Dairy Sci.

[70] Zhao FQ, Miller PJ, Wall EH (2004). Bovine glucose transporter GLUT8: cloning, expression, and developmental regulation in mammary gland. Biochim Biophys Acta.

[71] Zhao FQ, Glimm DR, Kennelly JJ (1993). Distribution of mammalian facilitative glucose transporter messenger RNA in bovine tissues. Int J Biochem.

[72] Zhao K, Liu HY, Wang HF, Zhou MM, Liu JX (2012). Effect of glucose availability on glucose transport in bovine mammary epithelial cells. Animal.

[73] Xiao CT, Cant JP (2005). Relationship between glucose transport and metabolism in isolated bovine mammary epithelial cells. J Dairy Sci.

[74] Shao Y, Wall EH, McFadden TB (2013). Lactogenic hormones stimulate expression of lipogenic genes but not glucose transporters in bovine mammary gland. Domest Anim Endocrinol.

[75] Zhang JZ, Behrooz A, Ismail-Beigi F (1999). Regulation of glucose transport by hypoxia. Am J Kidney Dis.

